# Adverse perinatal outcomes in twins: comparison of intertwin fetal size discordance *vs* singleton and twin fetal growth charts

**DOI:** 10.1002/uog.70139

**Published:** 2025-12-04

**Authors:** V. Giorgione, M. Lopian, M. Trapani, M. Brutto, M. G. Ferrante, A. Bhide, J. C. Jani, D. A. Badr, T. Ghi, E. Bevilacqua, A. Familiari, B. Thilaganathan

**Affiliations:** ^1^ Fetal Medicine Unit, St George's University Hospitals NHS Foundation Trust London UK; ^2^ Department of Women and Child Health Women Health Area, Fondazione Policlinico Universitario Agostino Gemelli IRCCS Rome Italy; ^3^ Vascular Biology Research Centre Molecular and Clinical Sciences Research Institute, St George's University of London London UK; ^4^ Department of Obstetrics and Gynecology, University Hospital Brugmann Université Libre de Bruxelles Brussels Belgium; ^5^ Catholic University of the Sacred Heart Rome Italy

**Keywords:** adverse perinatal outcome, estimated fetal weight, fetal growth reference standards, fetal growth restriction, intertwin size discordance, middle cerebral artery, stillbirths, twins, umbilical artery

## Abstract

**Objective:**

To compare the predictive performance of intertwin estimated fetal weight (EFW) discordance and EFW centile calculated according to either the Fetal Medicine Foundation (FMF) singleton or twin‐specific fetal growth charts for adverse perinatal outcome in dichorionic and monochorionic twin pregnancies.

**Methods:**

This was a retrospective multicenter cohort study of twin pregnancies managed between January 2013 and December 2023 at three tertiary fetal medicine centers in the UK, Italy and Belgium. Twin pregnancies in which an obstetric ultrasound exam was performed to obtain fetal biometry within 2 weeks before live birth or diagnosis of intrauterine fetal demise of one or both twins were included. Cases with anastomotic complications were excluded. The primary outcome was a composite adverse perinatal outcome (CAPO), defined as stillbirth (intrauterine fetal demise ≥ 22 weeks) of at least one cotwin and/or iatrogenic early preterm birth (delivery < 34 weeks) for fetal indications. The predictive performance of intertwin EFW discordance and of the EFW centile of the smaller twin, calculated using either singleton or twin‐specific FMF fetal growth charts, was assessed for CAPO. Predictive models were developed using logistic regression analysis and evaluated using the area under the receiver‐operating‐characteristics curve (AUC); pairwise comparisons between models were performed using DeLong's test.

**Results:**

The study analyzed 1294 dichorionic and 487 monochorionic twin pregnancies. For the prediction of CAPO, intertwin EFW discordance in dichorionic twins achieved an AUC of 0.93 (95% CI, 0.89–0.98), compared with 0.83 (95% CI, 0.77–0.90) (*P* = 0.001) and 0.87 (95% CI, 0.81–0.93) (*P* = 0.017) for EFW centile based on singleton and twin‐specific growth charts, respectively. Likewise, intertwin EFW discordance achieved an AUC of 0.95 (95% CI, 0.92–0.97) for predicting CAPO in monochorionic twins, outperforming EFW centile based on singleton charts (AUC, 0.80 (95% CI, 0.73–0.87); *P* < 0.001) and twin‐specific growth charts (AUC, 0.83 (95% CI, 0.76–0.90); *P* < 0.001). In clinical terms, at a 20% false‐positive rate (FPR), the sensitivity for CAPO in dichorionic twin pregnancies was 74%, 81% and 93% using singleton charts, twin‐specific charts and intertwin EFW discordance, respectively. Similarly, in monochorionic twin pregnancies, the sensitivity at a FPR of 20% was 75%, 79% and 98% using singleton charts, twin‐specific charts and intertwin EFW discordance, respectively.

**Conclusions:**

Intertwin EFW discordance is a more reliable predictor of the composite outcome of stillbirth in at least one cotwin and/or iatrogenic early preterm birth in twin pregnancies than is EFW centile based on either singleton or twin‐specific growth charts. This approach addresses the limitations of using different fetal growth reference charts that rely on arbitrary cut‐offs. Following external validation, the use of intertwin EFW discordance prediction algorithms may potentially enhance risk stratification to improve clinical outcomes in twin pregnancies. © 2025 The Author(s). *Ultrasound in Obstetrics & Gynecology* published by John Wiley & Sons Ltd on behalf of International Society of Ultrasound in Obstetrics and Gynecology.

## INTRODUCTION

Twin pregnancies, whether monochorionic or dichorionic, offer a unique clinical challenge owing to their elevated risk of adverse perinatal outcomes, such as preterm birth (PTB) and perinatal mortality and morbidity^1^. Although the risk of adverse perinatal outcome is usually higher in twin pregnancies than in singleton pregnancies, the risk factors differ from those in singletons^2^. One major difference between singleton and twin pregnancies is the higher incidence of placental dysfunction and the related impact on fetal growth trajectories in twins^3^, ^4^, ^5^, ^6^. The growth of twins diverges from that of singletons in the third trimester, presumably owing to physiological and environmental factors as well as uteroplacental insufficiency. This divergence often leads to a larger proportion of twins being classified as small‐for‐gestational age (SGA) when singleton charts are used, potentially overdiagnosing SGA without clear evidence that this confers clinical benefit^7^. While singleton growth charts have been used traditionally as a benchmark, emerging evidence has highlighted a potential role of twin‐specific growth charts to monitor twin growth^8^. However, there is an ongoing debate over whether twin‐specific growth charts appropriately distinguish physiological adaptation or inadvertently normalize pathological growth restriction compared with singleton charts^9^.

An alternative approach to identify fetuses at risk of adverse perinatal outcome, independent of fetal size as defined by different growth charts, is to assess estimated fetal weight (EFW) discordance between twins. With this approach, the larger twin is used as an internal pregnancy control to assess the degree of intertwin growth discordance, irrespective of gestational age. A significant intertwin size discordance is a well‐recognized predictor of adverse perinatal outcome^10^, ^11^, ^12^. Unlike SGA classification, which relies on absolute fetal size and can vary significantly depending on the different growth charts used, intertwin EFW discordance accounts for the relative growth differences between the twins, potentially offering valuable clinical insights into growth abnormalities.

This study aimed to compare the predictive performance of intertwin EFW discordance and EFW centile defined by the Fetal Medicine Foundation (FMF) singleton or twin‐specific fetal growth charts for adverse perinatal outcomes in dichorionic and monochorionic twin pregnancies.

## METHODS

This retrospective cohort study was conducted at three tertiary fetal medicine centers across Europe: St George's Hospital in London (UK), Fondazione Policlinico Universitario Agostino Gemelli IRCCS in Rome (Italy) and University Hospital Brugmann in Brussels (Belgium). The study examined twin pregnancies managed between January 2013 and December 2023 in these three centers. Ethical approval was not required, as fully anonymized retrospective clinical data were used, complying with UK, Italian or Belgian regulations. In this study, the sample size was determined by the number of eligible cases present in the datasets from the three participating centers, rather than being predefined using formal sample size calculations.

Eligible for inclusion were twin pregnancies of known chorionicity with a confirmed birth outcome, in which an obstetric ultrasound scan to obtain fetal biometry was performed within 2 weeks before live birth or diagnosis of intrauterine fetal demise of one or both twins. Pregnancies were excluded if they were affected by structural anomaly, chromosomal abnormality or miscarriage (up to 21 + 6 weeks' gestation). Monoamniotic monochorionic pregnancies and pregnancies with anastomotic complications, such as twin–twin transfusion syndrome or twin anemia–polycythemia sequence, were also excluded. Chorionicity and gestational age were determined by first‐trimester ultrasound assessment.

Case identification was carried out via a retrospective review of ultrasound databases in each location, and maternal demographic data, pregnancy characteristics and outcomes were extracted from the electronic ultrasound records and medical files. Twins were categorized based on EFW, with the smaller twin designated as Twin 1 and the larger one as Twin 2. EFW was calculated using the Hadlock formula^13^, which incorporates head circumference, abdominal circumference and femur length, and EFW centiles were calculated using singleton and twin‐specific growth reference charts from the FMF^6^, ^13^, ^14^. Both charts are based on ultrasound‐derived fetal biometry and reflect *in‐utero* growth, not neonatal birth weight. SGA was defined as EFW < 10^th^ centile. Discordance in EFW measurements was calculated as:

$$\begin{array}{ll} \mathrm{EFW}\;\text{discordance}\;(\%) & =(\text{higher}\;\mathrm{EFW}-\text{lower}\;\mathrm{EFW})/\\ & \;\text{higher}\;\mathrm{EFW}\times 100. \end{array}$$

All three referral centers adhered to the updated International Society of Ultrasound in Obstetrics and Gynecology (ISUOG) practice guidelines on the management of twin pregnancies, employing standardized Doppler surveillance of the umbilical artery, fetal middle cerebral artery and ductus venosus (when indicated). In uncomplicated monochorionic pregnancies, assessments were performed every 2 weeks starting from 16 weeks' gestation, while in uncomplicated dichorionic pregnancies, Doppler evaluation was conducted every 4 weeks from 20 weeks' gestation. In cases of fetal growth restriction, the frequency of Doppler assessments was increased, with evaluations performed at least once a week in monochorionic pregnancies and approximately every 2 weeks in dichorionic pregnancies, depending on the severity of the condition^15^.

The primary outcome was a composite adverse perinatal outcome (CAPO), defined as stillbirth of at least one cotwin (intrauterine fetal demise ≥ 22 weeks) and/or iatrogenic early PTB (delivery < 34 weeks) performed to prevent a perinatal loss in the case of indications such as abnormal findings on ultrasound examination, Doppler assessment or cardiotocography. Iatrogenic early PTB performed for fetal indications was classified as an adverse outcome to mitigate the impact of intervention bias of clinical interventions to prevent perinatal loss.

### Statistical analysis

Data were assessed for normality using visual inspection of histograms. Categorical variables are expressed as *n* (%) and continuous variables are presented as median (interquartile range (IQR)). Binomial logistic regression models were used to investigate the associations of intertwin EFW discordance adjusted for gestational age, SGA defined as EFW < 10^th^ centile (categorical) and EFW centile (continuous) of the smaller twin with CAPO. EFW data for each cotwin was missing in less than 5% of cases. Receiver‐operating‐characteristics (ROC) curves were generated to assess the associations between, and the predictive ability of, key variables for CAPO, with results presented as the area under the ROC curve (AUC) with 95% CI. Pairwise comparisons of AUCs from models using continuous variables were performed using DeLong's test. ROC curves were used to estimate the sensitivities of intertwin EFW discordance and the EFW centiles of the smaller twin, derived from both singleton and twin‐specific fetal growth charts, at fixed false‐positive rates (FPRs). Subgroup analysis was performed separately for dichorionic and monochorionic twin pregnancies. All statistical analysis was conducted using SPSS version 28.0 (SPSS Inc., Armonk, NY, USA) and R version 4.5 (R Foundation for Statistical Computing, Vienna, Austria). *P* < 0.05 was considered statistically significant.

## RESULTS

The study cohort included a total of 1781 twin pregnancies, of which 1294 were dichorionic and 487 were monochorionic. Baseline maternal demographic characteristics and delivery data of the entire cohort are presented in Table 1. Stillbirth of at least one cotwin or iatrogenic early PTB for fetal indications occurred in 58 (4.5%) dichorionic and 57 (11.7%) monochorionic twin pregnancies. Among pregnancies in which stillbirth occurred, iatrogenic early PTB also occurred in 3/15 dichorionic and 2/16 monochorionic pregnancies.

**Table 1:** Maternal demographic characteristics and delivery data of twin pregnancies with confirmed birth outcome and ultrasound performed within 2 weeks before live birth or stillbirth (*n* = 1781)

Characteristic	Value
Maternal age (years)	33 (29–37)
Prepregnancy BMI (kg/m^2^)	25 (22–29)
Non‐White ethnicity	359/1560 (23.0)
Smoker	89/1772 (5.0)
Alcohol consumption prior to conception	29/1615 (1.8)
Assisted conception	313/1736 (18.0)
Interval between ultrasound and delivery (days)	8 (5–12)
Gestational age at delivery (weeks)	36.3 (34.6–37.1)
Birth‐weight centile (Twin 1)	2.1 (0.2–10.1)‡
SGA at birth (Twin 1)	1302/1740 (74.8)
Birth‐weight centile (Twin 2)	17.2 (5.4–36.7)§
SGA at birth (Twin 2)	638/1757 (36.3)
Stillbirth*	
Dichorionic twins	15/1294 (1.2)
Monochorionic twins	16/487 (3.3)
Iatrogenic early preterm birth†	
Dichorionic twins	43/1294 (3.3)
Monochorionic twins	41/487 (8.4)

Data are presented as median (interquartile range) or *n*/*N* (%). Twin 1 refers to smaller twin and Twin 2 is the larger twin. Less than 10% of data were missing for maternal age, body mass index (BMI), interval between ultrasound and delivery, and gestational age at delivery.

* Intrauterine fetal death ≥ 22 weeks of at least one cotwin.

† Delivery < 34 weeks for fetal indications.

‡ Data are missing in 41 cases.

§ Data are missing in 24 cases. SGA, small‐for‐gestational age.

**Figure 1:**
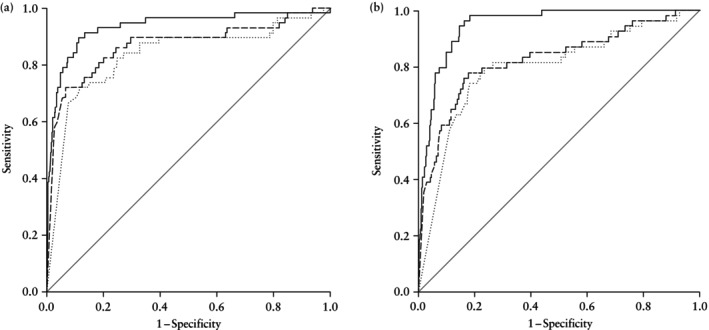
Receiver‐operating‐characteristics curves for predictive models for composite adverse perinatal outcome of stillbirth of at least one cotwin and/or iatrogenic early preterm birth < 34 weeks based on intertwin estimated fetal weight (EFW) discordance (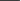) and EFW centile of smaller twin based on singleton (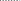) or twin (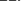) fetal growth charts in dichorionic (a) and monochorionic (b) twin pregnancies.

In the dichorionic cohort, use of the singleton or twin‐specific fetal growth charts resulted in a diagnosis of SGA in 765 (59.1%) and 504 (38.9%) pregnancies, respectively. The median intertwin EFW discordance was 8.6% (IQR, 3.9–15.6%). Intertwin EFW discordance adjusted for gestational age was strongly correlated with CAPO, with an adjusted odds ratio (aOR) of 1.10 (95% CI, 1.07–1.12) and an AUC of 0.93 (95% CI, 0.89–0.98). Intertwin EFW discordance demonstrated superior predictive performance, with a higher AUC for CAPO compared with SGA diagnosis of the smaller twin based on either singleton or twin growth charts, irrespective of whether a categorical (SGA < 10^th^ centile) or continuous (EFW centile) variable was analyzed (Table 2, Figure 1a). For example, using the model of SGA < 10^th^ centile defined by the twin‐specific fetal growth chart resulted in an AUC of 0.76 (95% CI, 0.71–0.81). Table 3 illustrates that the intertwin EFW discordance model showed a significantly better predictive performance for CAPO, with a greater AUC compared with both singleton (*P* = 0.001) and twin (*P* = 0.017) chart‐based SGA classifications.

**Table 2:** Performance of predictive models for stillbirth of at least one cotwin and/or iatrogenic early preterm birth < 34 weeks based on singleton or twin fetal growth charts or intertwin estimated fetal weight (EFW) discordance in dichorionic and monochorionic twin pregnancies

Model	OR (95% CI)	AUC (95% CI)
*Dichorionic twins*		
SGA < 10^th^ centile (singleton chart)*	5.83 (2.48–13.70)	0.65 (0.59–0.71)
SGA < 10^th^ centile (twin chart)*	14.09 (6.00–33.10)	0.76 (0.71–0.81)
EFW centile (singleton chart)†	0.95 (0.92–0.98)	0.83 (0.77–0.90)
EFW centile (twin chart)†	0.94 (0.91–0.96)	0.87 (0.81–0.93)
Intertwin EFW discordance†, ‡	1.10 (1.07–1.12)	0.93 (0.89–0.98)
*Monochorionic twins*		
SGA < 10^th^ centile (singleton chart)*	4.44 (1.96–10.04)	0.63 (0.56–0.70)
SGA < 10^th^ centile (twin chart)*	10.47 (5.24–20.93)	0.76 (0.70–0.83)
EFW centile (singleton chart)†	0.94 (0.91–0.98)	0.80 (0.73–0.87)
EFW centile (twin chart)†	0.95 (0.93–0.97)	0.83 (0.76–0.90)
Intertwin EFW discordance†, ‡	1.10 (1.07–1.14)	0.95 (0.92–0.97)

* Categorical models for small‐for‐gestational‐age (SGA) diagnosis based on EFW < 10^th^ centile.

† Continuous models.

‡ EFW discordance was adjusted for gestational age. AUC, area under the receiver‐operating‐characteristics curve; OR, odds ratio.

**Table 3:** Comparison of predictive performance of models for stillbirth of at least one cotwin and/or iatrogenic early preterm birth < 34 weeks based on estimated fetal weight (EFW) centile of smaller twin calculated using singleton or twin fetal growth charts or intertwin EFW discordance in dichorionic and monochorionic twin pregnancies

Comparison of models*	AUC of model 1	AUC of model 2	ΔAUC (95% CI)	*P*
*Dichorionic twins*				
EFW centile (twin chart) *vs* EFW centile (singleton chart)	0.87	0.83	0.04 (0.02–0.05)	< 0.001
Intertwin EFW discordance *vs* EFW centile (singleton chart)	0.93	0.83	0.10 (0.04–0.16)	0.001
Intertwin EFW discordance *vs* EFW centile (twin chart)	0.93	0.87	0.06 (0.01–0.12)	0.017
*Monochorionic twins*				
EFW centile (twin chart) *vs* EFW centile (singleton chart)	0.83	0.80	0.03 (0.02–0.04)	< 0.001
Intertwin EFW discordance *vs* EFW centile (singleton chart)	0.95	0.80	0.15 (0.09–0.21)	< 0.001
Intertwin EFW discordance *vs* EFW centile (twin chart)	0.95	0.83	0.12 (0.06–0.18)	< 0.001

* Comparison is model 1 *vs* model 2 for each row. AUC, area under the receiver‐operating‐characteristics curve; ΔAUC, difference in AUC.

In the monochorionic cohort, use of the singleton or twin‐specific fetal growth charts resulted in a diagnosis of SGA in 306 (62.8%) and 163 (33.5%) pregnancies, respectively. The median intertwin EFW discordance was 9.7% (IQR, 4.6–17.8%). Intertwin EFW discordance adjusted for gestational age was strongly correlated with CAPO, with an aOR of 1.10 (95% CI, 1.07–1.14) and an AUC of 0.95 (95% CI, 0.92–0.97). Intertwin EFW discordance demonstrated superior predictive performance, with a higher AUC for CAPO compared with SGA diagnosis of the smaller twin based on either singleton or twin growth charts, irrespective of whether a categorical (SGA < 10^th^ centile) or continuous (EFW centile) variable was analyzed (Table 2, Figure 1b). For example, the model of SGA < 10th centile defined by the twin growth chart had an AUC of 0.76 (95% CI, 0.70–0.83). The intertwin EFW discordance model showed significantly better predictive performance for CAPO compared with the singleton (*P* < 0.001) and twin (*P* < 0.001) growth charts (Table 3).

The sensitivities of intertwin EFW discordance and EFW centiles of the smaller twin, calculated using singleton or twin‐specific fetal growth charts, at various FPRs in dichorionic and monochorionic twin pregnancies are shown in Table 4. The relative cut‐offs of intertwin EFW discordance and EFW centile according to singleton and twin charts are presented in Table S1, showing that at a 20% FPR the intertwin EFW discordance threshold was 15% for both monochorionic and dichorionic twin pregnancies.

**Table 4:** Predictive performance of models for stillbirth of at least one cotwin and/or iatrogenic early preterm birth < 34 weeks based on estimated fetal weight (EFW) centile of smaller twin calculated using singleton or twin fetal growth charts or intertwin EFW discordance in dichorionic and monochorionic twin pregnancies at various fixed false positive rates (FPRs)

Model	5% FPR	10% FPR	20% FPR	40% FPR
*Dichorionic twins*				
EFW centile (singleton chart)	0.67 (0.54–0.80)	0.68 (0.55–0.81)	0.74 (0.62–0.86)	0.90 (0.83–0.97)
EFW centile (twin chart)	0.67 (0.54–0.80)	0.72 (0.60–0.84)	0.81 (0.71–0.91)	0.90 (0.83–0.97)
Intertwin EFW discordance	0.77 (0.66–0.88)	0.84 (0.74–0.94)	0.93 (0.86–0.99)	0.97 (0.93–1.00)
*Monochorionic twins*				
EFW centile (singleton chart)	0.40 (0.28–0.53)	0.59 (0.46–0.72)	0.75 (0.64–0.86)	0.82 (0.72–0.92)
EFW centile (twin chart)	0.45 (0.32–0.58)	0.61 (0.47–0.75)	0.79 (0.68–0.90)	0.84 (0.74–0.94)
Intertwin EFW discordance	0.65 (0.52–0.78)	0.85 (0.76–0.95)	0.98 (0.94–1.02)	0.98 (0.94–1.02)

Data are given as sensitivity (95% CI).

## DISCUSSION

This study demonstrates the value of intertwin EFW discordance in optimizing risk stratification for adverse perinatal outcomes in twin pregnancies. In both dichorionic and monochorionic pregnancies, intertwin EFW discordance was more strongly associated with the prediction of CAPO than SGA classification based on either FMF singleton or twin fetal growth charts, irrespective of whether fetal size was analyzed as a categorical (SGA < 10^th^ centile) or as a continuous (EFW centile) variable.

### SGA diagnosed using singleton and twin growth charts

The use of singleton growth charts for twins results in the identification of a greater proportion of fetuses with growth restriction owing to placental insufficiency or other pathological conditions. Singleton growth charts have demonstrated higher sensitivity for detecting growth‐restricted twins at risk of adverse perinatal outcome^9^. However, the main limitation is the tendency of singleton charts to classify a large proportion of normally growing twins as SGA, with rates reaching up to 50%^8^, ^16^. The overdiagnosis of SGA increases the risk of unnecessary iatrogenic preterm delivery and other interventions, such as corticosteroid administration and frequent hospital visits. Twin‐specific growth charts have demonstrated improved specificity for identifying twins at increased risk of adverse perinatal outcome, thereby reducing the prevalence of false‐positive SGA diagnoses^17^. As a consequence, studies have shown that twins classified as SGA using twin‐specific fetal growth charts have a greater association with adverse perinatal outcome than those identified using singleton charts, but usually with a reduced detection rate^8^, ^16^, ^18^. Additionally, twin‐specific charts may reduce maternal stress and healthcare costs by minimizing unnecessary interventions^8^. However, concerns remain that the reduced detection rate of adverse perinatal outcome associated with use of twin‐specific charts is a consequence of the normalization of pathological growth restriction, leading to missed diagnoses of fetuses that require closer surveillance.

### Intertwin EFW discordance

Intertwin size discrepancy, measured as EFW discordance, offers a practical approach to overcome the controversy surrounding the use of singleton or twin growth charts in evaluating fetal wellbeing in twin pregnancies. Intertwin EFW discordance circumvents the limitations of using fetal growth charts by assessing the relative growth difference between twins rather than comparing each fetus with a growth reference. This measure identifies cases in which one fetus lags significantly behind the growth of its cotwin, suggesting an increased risk of placental insufficiency and a reduced likelihood of physiological smallness. Consistent with this hypothesis, we observed significantly improved prediction of intrauterine fetal demise or the need for iatrogenic early PTB to prevent perinatal loss when using intertwin EFW discordance compared with singleton or twin‐specific fetal growth charts. For example, comparison of the various methods for assessing placental dysfunction in dichorionic twins demonstrated that at a 20% FPR, the sensitivity for CAPO was 74%, 81% and 93% using singleton charts, twin charts or intertwin EFW discordance, respectively (Table 4). Even when very high FPRs of ≥ 40% are achieved, intertwin EFW discordance still maintains an advantage over the use of singleton or twin‐specific charts for the identification of high‐risk pregnancies.

Despite the strong performance of intertwin EFW discordance in predicting CAPO in our study, these findings must be interpreted within the context of certain limitations. The higher AUCs observed may, in part, reflect the inclusion of iatrogenic early PTB as part of a composite outcome, which could be influenced by clinician decisions rather than spontaneous deterioration. Furthermore, while intertwin EFW discordance outperformed singleton and twin‐specific fetal growth chart approaches, this variable may be less effective in predicting risk in twin pairs in which both fetuses are equally small or large. Finally, although statistical differences between models were significant, the clinical relevance of modest differences in AUC values should be weighed carefully when translating findings into practice.

### Research and clinical implications

The findings of this study pave the way for future research to optimize growth‐monitoring protocols in twin pregnancies. Prospective studies are needed to externally validate these findings across larger, diverse cohorts and evaluate whether using an algorithm based on intertwin EFW discordance in routine clinical practice can improve perinatal outcomes. Furthermore, Doppler studies in twin pregnancies have shown that abnormal umbilical artery Doppler findings, such as increased pulsatility index or absent/reversed end‐diastolic flow, are strongly associated with increased neonatal risks^19^. Therefore, future studies should also investigate the role of additional Doppler parameters measured throughout pregnancy in refining risk prediction.

### Strengths and limitations

The strengths of this study include its large cohort size and the inclusion of data from three tertiary fetal medicine centers across Europe, which enhances the generalizability of the findings. The robust methodology of the study, including standardized ultrasound protocols and comprehensive statistical analysis, adds further validity to the results. However, the retrospective design introduced potential bias, such as incomplete data or selection bias, which may impact the findings. Additionally, while the study includes data from several centers, differences in clinical practice or population characteristics across sites could also have influenced the results. The potential for intervention bias, whereby variations in clinical management based on local protocols or physician preferences could have affected outcomes, is another limitation. The relatively high prevalence of SGA observed in our cohort may reflect the referral nature of the participating centers, which are more likely to manage high‐risk pregnancies. As such, the study population may not fully represent the general population of twin pregnancies. Finally, the study did not account for certain maternal factors, such as pre‐existing comorbidities or other obstetric complications such as pre‐eclampsia or gestational diabetes, which may influence fetal growth and Doppler findings.

### Conclusions

This study highlights that intertwin EFW discordance is a more robust predictor of adverse perinatal outcome than is SGA classification using either singleton or twin‐specific fetal growth charts. These results emphasize the need to incorporate intertwin EFW discordance into routine monitoring protocols for twin pregnancies. By adopting a comprehensive, multifaceted approach that utilizes growth parameters as continuous variables rather than relying on arbitrary cut‐off values, clinicians could improve the detection of fetuses at risk of adverse perinatal outcome. This strategy may enhance perinatal care, reduce morbidity and lower mortality rates in twin pregnancies.

## Supporting information


**Table S1** Cut‐off values for intertwin estimated fetal weight (EFW) discordance and EFW centile based on singleton and twin growth charts at fixed false‐positive rates (FPRs) for prediction of composite adverse perinatal outcome in dichorionic and monochorionic twin pregnancies.

## Data Availability

The data that support the findings of this study are available from the corresponding author upon reasonable request.
