# Supplementary material for: Adverse perinatal outcomes in twins: comparison of intertwin fetal size discordance vs singleton and twin fetal growth charts
Source: Ultrasound Obstet Gynecol. 2025 Dec 4;67(1):42–8. doi: 10.1002/uog.70139 (PMC12757814; doi:10.1002/uog.70139)
Supplement: Supplementary file 1 — Table S1 Cut‐off values for intertwin estimated fetal weight (EFW) discordance and EFW centile based on singleton and twin growth charts at fixed false‐positive rates (FPRs) for prediction of composite adverse perinatal outcome in dichorionic and monochorionic twin pregnancies. [file UOG-67-42-s001.docx]

**Supplementary Table 1** Cut-off values for estimated fetal weight (EFW) discordance and EFW centile based on singleton and twin growth charts at fixed false-positive rates (FPR) for prediction of composite adverse perinatal outcome in dichorionic and monochorionic twin pregnancies

|  | **Cut-offs**  **5% FPR** | **Cut-offs**  **10% FPR** | **Cut-offs**  **20% FPR** | **Cut-offs**  **40% FPR** |
| --- | --- | --- | --- | --- |
| **Dichorionic twins** | | | | |
| EFW discordance | 30 | 22 | 15 | 10 |
| Singleton chart estimated fetal weight centile | 1.25 | 2.65 | 4.45 | 8.05 |
| Twin chart estimated fetal weight centile | 0.9 | 2.15 | 4.45 | 8.05 |
| **Monochorionic twins** | | | | |
| EFW discordance | 25 | 21 | 15 | 10 |
| Singleton chart estimated fetal weight centile | 1.5 | 3 | 4.9 | 8.6 |
| Twin chart estimated fetal weight centile | 1.3 | 2.6 | 4.3 | 8 |

This table shows the threshold values for estimated fetal weight (EFW) discordance and estimated fetal weight centiles based on singleton and twin growth charts, corresponding to fixed false positive rates (FPRs) of 5%, 10%, 20%, and 40% for the prediction of adverse perinatal outcomes in dichorionic and monochorionic twins. EFW discordance values are presented as percentages, while centiles are expressed numerically.
